# Decoding Small Cell Lung Cancer: Molecular Subtypes, Surface Antigens, and the Target-Modality Problem

**DOI:** 10.3390/cancers18132173

**Published:** 2026-07-07

**Authors:** Mijail I. Zambrano Iglesias, Daniel Rosas, Salih Akgun, Ines C. Padron Cubillan, Fedor Wadi Richani Meinhardt, Atif Hussein, Luis E. Raez

**Affiliations:** 1 Memorial Cancer Institute, Pembroke Pines, FL 33026, USA; drosas@mhs.net (D.R.); ipadroncubillan@mhs.net (I.C.P.C.); frichanimeinhardt@mhs.net (F.W.R.M.); ahussein@mhs.net (A.H.); lraez@mhs.net (L.E.R.); 2 Hackensack Meridian Health, JFK University Medical Center, Edison, NJ 08820, USA; md.salihakgun@gmail.com; 3 Institute for Human Health and Disease Intervention, Florida Atlantic University (FAU), Jupiter, FL 33458, USA

**Keywords:** small cell lung cancer, transcriptomic subtypes, ASCL1, NEUROD1, POU2F3, DLL3, tarlatamab, bispecific T-cell engager, B7-H3, precision oncology

## Abstract

Small cell lung cancer (SCLC) has historically been treated as a single, uniformly aggressive disease defined by neuroendocrine differentiation, near-universal loss of TP53 and RB1, and the absence of classical druggable oncogene addictions. Two converging lines of evidence are now reshaping that view. First, transcriptomic profiling has resolved SCLC into molecular subtypes—SCLC-A (ASCL1-driven), SCLC-N (NEUROD1-driven), SCLC-P (POU2F3-driven), and SCLC-I (inflamed)—with distinct immune microenvironments, surface-antigen expression patterns, and emerging therapeutic vulnerabilities, although intratumoral heterogeneity and phenotypic plasticity complicate clean subtype assignment. Second, the development of delta-like ligand 3 (DLL3)-directed therapies provides a natural experiment: the same validated surface antigen failed as an antibody–drug conjugate (rovalpituzumab tesirine, three negative randomized trials) yet succeeded as a bispecific T-cell engager (tarlatamab, which received FDA accelerated approval in 2024 and subsequent traditional FDA approval in 2025 following positive confirmatory phase 3 data). In this review, we integrate the current first-line standard of care—chemoimmunotherapy with atezolizumab- or durvalumab-based regimens followed by maintenance intensification with lurbinectedin–atezolizumab (IMforte)—with the molecular framework of subtypes and biomarkers, and we use DLL3 as a case study to propose that delivery modality is an important determinant of therapeutic success in SCLC and should be considered alongside target biology and tumor heterogeneity. Rapid proliferation, antigen heterogeneity, subtype plasticity, and a relatively less immunogenic microenvironment systematically penalize modalities dependent on payload accumulation and cell-cycle progression and reward modalities that recruit catalytic, cell-cycle-independent cytotoxic effectors. The emerging B7-H3 and SEZ6 programs—including ifinatamab deruxtecan and ABBV-706—are the next test of this framework. We discuss implications for biomarker development, trial design, and the operational challenges of subtype-guided precision oncology in a disease where tissue is scarce and biology shifts under therapy.

## Simple Summary

Small cell lung cancer (SCLC) remains one of the most aggressive thoracic malignancies, with limited long-term survival despite recent therapeutic advances. This review summarizes the evolving molecular classification of SCLC, emerging predictive biomarkers, and the development of surface-antigen-directed therapies such as DLL3, B7-H3, and SEZ6-targeted agents. We highlight how therapeutic modality selection may determine clinical success, as demonstrated by the contrasting outcomes of DLL3-targeted antibody–drug conjugates and bispecific T-cell engagers. These findings support a subtype-guided precision-oncology framework and may help guide future therapeutic strategies in SCLC. Furthermore, ongoing translational research and biomarker-driven therapeutic development may facilitate more personalized treatment approaches and improve clinical outcomes for patients with this challenging disease.

## 1. Introduction

Small cell lung cancer (SCLC) accounts for approximately 13–15% of lung cancers and is among the most chemosensitive yet ultimately lethal solid tumors, with extensive-stage (ES-SCLC) five-year survival historically below 7% [1,2]. The molecular biology of SCLC has been understood in broad outline for nearly a decade: near-universal biallelic loss of TP53 and RB1, frequent amplification of MYC paralogs, and an absence of the canonical actionable oncogenic drivers (EGFR, ALK, ROS1, KRAS G12C) that have transformed non-small cell lung cancer (NSCLC) management [3,4]. Nevertheless, recurrent alterations involving MYC, BCL-2, and NOTCH signaling identify biologically relevant vulnerabilities that are actively being explored therapeutically, although none currently has an FDA-approved targeted therapy in SCLC [4,5,6,7]. Therapy has therefore leaned on cytotoxic chemotherapy and, since 2018–2019, on the addition of Programmed Death-Ligand 1 (PD-L1) inhibitors to platinum–etoposide induction, with modest gains in median survival but a meaningful tail of long-term survivors [8,9].

This apparent paradox reflects the biology of SCLC: despite high initial chemosensitivity, most tumors rapidly develop resistance, resulting in short progression-free intervals and ultimately contributing to one of the highest mortality rates among solid malignancies [1,2,10].

Three developments have, in our view, shifted the field. First, the IMforte trial established maintenance intensification with lurbinectedin plus atezolizumab as a new option after chemoimmunotherapy induction, reframing ES-SCLC as a disease requiring continuous therapeutic pressure rather than fixed-duration treatment [11]. Second, transcriptomic profiling has resolved SCLC into four molecular subtypes—SCLC-A, SCLC-N, SCLC-P, and SCLC-I—with distinct lineage-defining transcription factor programs, immune microenvironments, and surface-antigen expression patterns [12,13]. Third, the clinical development of delta-like ligand 3 (DLL3) has produced an instructive natural experiment: the same validated surface antigen, pursued by two distinct delivery modalities, has produced opposite clinical outcomes—failure for an antibody–drug conjugate (ADC) [14,15,16,17] and approval for a Bispecific T-cell Engager (BiTE) [18,19,20,21].

These three developments are usually discussed in isolation. We argue they cohere into a single framework. The clinical motivation for molecular stratification comes from the limits of current chemoimmunotherapy and the absence of validated predictive biomarkers in the IMpower133, CASPIAN, and IMforte trials [11,22,23]. The molecular framework—subtypes, plasticity, and surface-antigen biology—provides the substrate for subtype-guided precision oncology. The DLL3 case study sharpens what target validation means in a disease without classical oncogene addiction and demonstrates that, once a target is biologically validated, modality choice becomes the decisive variable. We close by examining how this framework should shape the development of B7-H3- and SEZ6-directed therapies, and where the framework’s limits lie.

This narrative review was conducted through a comprehensive literature search of PubMed/MEDLINE and major international oncology conference proceedings, including the American Society of Clinical Oncology (ASCO), the European Society for Medical Oncology (ESMO), and the World Conference on Lung Cancer (WCLC). Searches included articles and conference presentations published through June 2026 using combinations of the terms “small cell lung cancer,” “SCLC molecular subtypes,” “DLL3,” “B7-H3,” “SEZ6,” “tarlatamab,” “ifinatamab deruxtecan,” “bispecific T-cell engager,” “antibody-drug conjugate,” “CAR-T,” and “predictive biomarkers.” Priority was given to prospective clinical trials, pivotal phase I–III studies, translational investigations, consensus classifications, and high-impact review articles. Additional references were identified through manual review of bibliographies from relevant publications.

## 2. Current First-Line Standard of Care: From Chemoimmunotherapy to Maintenance Intensification

For more than three decades, platinum–etoposide chemotherapy (EP) was the standard first-line regimen for ES-SCLC, producing initial response rates of approximately 60–65% but a median overall survival (OS) near 10 months and five-year survival under 5% [2,10]. The integration of immune checkpoint inhibitors (ICIs) targeting the PD-1/PD-L1 axis to platinum–etoposide—established by IMpower133 and CASPIAN—was the first major change in this paradigm in a generation [8,9]. Most recently, the IMforte trial extended the framework to maintenance intensification with lurbinectedin plus atezolizumab [11].

### 2.1. IMpower133

IMpower133 was a randomized, double-blind phase I/III trial that evaluated atezolizumab plus carboplatin and etoposide in 403 treatment-naïve patients with ES-SCLC [8]. Following four cycles of induction with atezolizumab or placebo plus EP, patients without progression continued on maintenance atezolizumab or placebo until progression. Median OS was 12.3 months in the atezolizumab arm versus 10.3 months in the control arm (hazard ratio [HR] 0.70; *p* = 0.007); median progression-free survival (PFS) was 5.2 versus 4.3 months (HR 0.77) [8]. Updated OS analyses confirmed the durability of benefit (HR 0.76) without a clear association between treatment effect and PD-L1 expression or blood tumor mutational burden (bTMB), reinforcing that response to immunotherapy in SCLC is not predicted by single measurable parameters [22].

The IMpower133/IMbrella, a five-year analysis, provided the first long-term survival data for first-line chemoimmunotherapy in ES-SCLC: approximately 12% of patients treated with atezolizumab plus EP remained alive at five years, substantially exceeding historical chemotherapy-only rates [24]. This long tail on the survival curve—a small but real subset of durable benefit—is the most distinctive feature of chemoimmunotherapy in SCLC, and its biological determinants remain poorly characterized.

### 2.2. CASPIAN

CASPIAN, an open-label phase 3 trial, randomized 805 patients with ES-SCLC to durvalumab plus EP, durvalumab plus tremelimumab plus EP, or EP alone [9]. Durvalumab plus EP improved median OS (13.0 vs. 10.3 months; HR 0.73), and updated three-year follow-up demonstrated OS rates of 17.6% in the durvalumab arm versus 5.8% in the EP arm—a marked increase in long-term survivors [23]. CASPIAN differed from IMpower133 in its open-label design, allowance of either cisplatin or carboplatin, and inclusion of patients with untreated asymptomatic brain metastases. Importantly, the addition of the CTLA-4 inhibitor tremelimumab did not produce a statistically significant survival improvement, suggesting that dual immune checkpoint blockade does not provide additional benefit in this disease context [23]. As in IMpower133, biomarker analyses failed to identify a reliable predictor of response—neither PD-L1 expression nor TMB was associated with OS benefit [10,22,25].

### 2.3. IMforte and Maintenance Intensification

Despite improvements with chemoimmunotherapy, most ES-SCLC patients progress within months of initiating treatment, underscoring the transient nature of immune activation produced by current induction strategies. IMforte addressed this by evaluating maintenance intensification with lurbinectedin—a selective inhibitor of oncogenic transcription—added to atezolizumab after standard induction with atezolizumab plus carboplatin and etoposide [11]. Patients without progression after four cycles of induction were randomized to maintenance atezolizumab plus lurbinectedin or atezolizumab alone.

Both primary endpoints were met. Median PFS was 5.4 versus 2.1 months (HR 0.54), and median OS from the start of maintenance was 13.2 versus 10.6 months (HR 0.73; *p* = 0.0174) [11]. Lurbinectedin’s mechanism is dual: cytotoxic inhibition of oncogenic transcription in tumor cells and immunomodulatory depletion of tumor-associated macrophages with reshaping of the tumor microenvironment, providing a rationale for combination with PD-L1 blockade [11]. Several caveats temper interpretation. Outcomes were measured from the start of maintenance, not from initiation of treatment, and patients who progressed during induction were excluded from randomization, enriching the IMforte population for more favorable disease biology. Direct comparisons with IMpower133 and CASPIAN should therefore be made with caution. Even so, IMforte has demonstrated for the first time that post-induction therapeutic intensification can extend disease control in a disease whose dominant clinical pattern is rapid relapse.

### 2.4. The Biomarker Gap

A consistent finding across IMpower133, CASPIAN, and IMforte is the absence of validated predictive biomarkers for chemoimmunotherapy benefit [22,23,25]. PD-L1 expression on tumor cells in SCLC is typically low or absent (5–17% of tumors) and has not predicted benefit in any pivotal trial [25]. Tumor mutational burden showed initial promise in CheckMate 032 but was not validated as predictive in subsequent prospective studies, and bTMB was not predictive in IMpower133 [22]. The biomarker gap has direct clinical consequences: it limits the ability to identify patients most likely to benefit from immunotherapy, to spare those unlikely to benefit, or to design rational combinations. It also provides the strongest motivation for the molecular-subtype framework discussed in the next section. Trial designs and outcomes are summarized in Table 1.

Additional studies continue to refine therapeutic strategies in SCLC. IMpower133 established chemoimmunotherapy as the first-line standard (mOS 12.3 months; mPFS 5.2 months), while CheckMate-032 suggested improved outcomes with immunotherapy in patients with high tumor mutational burden. Emerging studies, including IDeate-Lung02, ARTEMIS-08, STRATUS, and EVOKE-SCLC-04, are evaluating B7-H3- and Trop-2-directed strategies and may further expand the biomarker-driven therapeutic landscape of SCLC [8,22,23,25,26,27].

## 3. Second Line Options and Beyond

### 3.1. Topotecan as a Historical Second-Line Standard

Topotecan, the first FDA-approved and most widely used second-line therapy for SCLC, is a topoisomerase I (TOP1) inhibitor that disrupts DNA replication in rapidly proliferating tumor cells, leading to apoptosis [28,29]. Thoroughly studied throughout the years, topotecan has demonstrated limited efficacy with a questionable safety profile. In a pivotal Phase III trial, this TOP1 inhibitor demonstrated superiority over best supportive care alone, establishing it as the second-line treatment of choice after chemotherapy progression. The study reported significant improvement in OS (25.9 weeks, vs. 13.9 weeks), a partial response rate of 7%, and 44% stable disease [28,30]. Despite these outcomes, significant findings of hematological toxicities such as grade 4 neutropenia, grade 4 thrombocytopenia, and grade 3/4 anemia raised concern, as frequent dose reduction or treatment interruptions were required. Nevertheless, patients receiving topotecan experienced slower quality of life deterioration and improved symptom control. Comparisons with other TOP1 inhibitors have yielded mixed results, though median OS favored topotecan (8.3 months) over the liposomal irinotecan (7.9 months) [31]. Additional trials have evaluated the efficacy of multiple platinum-based chemotherapy regimens, such as triplet therapy (cisplatin, etoposide, and irinotecan) over topotecan as a second-line in sensitive relapsed SCLC, and have shown improved outcomes; however, the toxicity burden continues to outweigh the potential benefits [32].

### 3.2. Lurbinectedin in Relapsed SCLC

More than two decades after the approval of topotecan, Lurbinectedin became the first agent to receive accelerated approval for the treatment of relapsed SCLC following progression on platinum-based chemotherapy [29,33]. This selective RNA Polymerase II inhibitor suppresses oncogenic transcription, modulates the tumor microenvironment by targeting tumor-associated macrophages, and reduces inflammatory chemokines (such as CCL2), ultimately promoting tumor-cell apoptosis [29]. In a phase II trial, Lurbinectedin emerged as a promising therapy, demonstrating an ORR of 35.2%, a PFS of 3.5 months, and a median OS of 9.3 months [28]. Preliminary reports of a phase I/II evaluating lurbinectedin in combination with irinotecan showed an ORR of 42.6%, a median DoR of 7.6 months, and a OS rate at 12 months of 44.6% [34]. These results supported the development of the phase III LAGOON trial evaluating lurbinectedin alone or in combination with irinotecan versus investigator’s choice of topotecan or irinotecan in relapsed SCLC. The study has completed enrollment and conduct; however, efficacy results have not yet been reported in the peer-reviewed literature and remain awaited [35].

## 4. Molecular Subtypes of SCLC: Beyond the One-Disease Model

### 4.1. The Four-Subtype Framework

**Figure 1:**
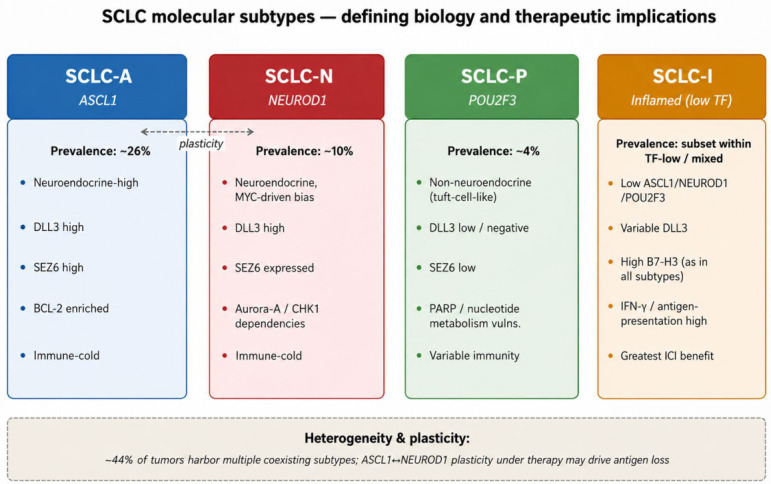
Molecular subtype framework of SCLC. The four-subtype model defines tumors by dominant lineage transcription factor expression: SCLC-A (ASCL1), SCLC-N (NEUROD1), SCLC-P (POU2F3), and SCLC-I (inflamed; low TF expression with an immune-active gene signature). Surface-antigen distribution differs systematically across subtypes—DLL3 and SEZ6 show the highest expression in ASCL1- and NEUROD1-driven tumors, although detectable DLL3 expression is observed across the majority of SCLC cases overall. Whereas B7-H3 is broadly expressed across all four subtypes. Approximately 44% of tumors harbor multiple coexisting subtypes, and ASCL1↔NEUROD1 plasticity under therapeutic pressure may drive antigen loss for surface-antigen-directed therapies.

The Rudin et al. consensus framework, proposed in 2019, classified SCLC into four subtypes based on the dominant expression of lineage-defining transcription factors: ASCL1 (SCLC-A), NEUROD1 (SCLC-N), POU2F3 (SCLC-P), and YAP1 (SCLC-Y) [17]. Gay et al. subsequently refined this framework using non-negative matrix factorization of tumor expression data, replacing SCLC-Y with SCLC-I (inflamed)—a subtype defined by low expression of all three lineage transcription factors accompanied by an inflamed gene signature, and predicted to derive the greatest benefit from chemoimmunotherapy [13]. Real-world multiomics profiling of 944 SCLC tumors by Puri et al. found SCLC-A in 25.6%, SCLC-N in 10.2%, SCLC-Y in 12.5%, SCLC-P in 4.3%, transcription factor-negative tumors in 19.5%, and mixed subtypes in 27.9% of cases [36]. The high prevalence of mixed and transcription factor-negative tumors is itself a signal that the four-subtype framework, while useful, does not cleanly partition clinical samples. The defining biology, surface-antigen patterns, and therapeutic vulnerabilities of each subtype are summarized in Figure 1 and Table 2.

### 4.2. Heterogeneity and Phenotypic Plasticity

The principal challenge to subtype-based precision oncology in SCLC is intratumoral heterogeneity. Peressini et al. profiled multi-region tumor samples from 58 ES-SCLC patients and found that 44% had tumors with multiple coexisting transcriptional subtypes, with subtype-specific target expression often inconsistent within individual tumors [38]. Tumors from patients with long-term chemoimmunotherapy benefit (time to progression ≥ 12 months) shared an interferon-γ-dominated mRNA profile with enhanced antigen presentation capacity, whereas hypoxia and glycolytic pathway activation were associated with resistance—a finding that points toward functional immune signatures rather than clean subtype assignment as a more reliable predictor of benefit [38].

Subtype boundaries themselves are blurred. Takumida et al. demonstrated that ASCL1 and NEUROD1 are co-expressed in nearly half of SCLC cases, with SCLC-A/N double-positive tumors exhibiting intermediate transcriptional and epigenetic characteristics regulated partly through divergent NEUROD1- and ASCL1-driven programs [41]. NOTCH signaling drives neuroendocrine-to-non-neuroendocrine transitions that contribute to chemoresistance, immune modulation, and metastasis [42]. Together, these findings support a spectrum-based model of SCLC identity in which subtypes are better understood as dynamic cellular states than fixed identities.

Subtype dynamics under therapy are clinically consequential. Tumors can shift from ASCL1-high to NEUROD1-high phenotypes under therapeutic pressure, and lineage transitions toward less-neuroendocrine states are associated with chemoresistance [42]. A patient whose tumor undergoes subtype transition between diagnosis and progression may lose the very antigen targeted by their next-line therapy. This has direct implications for surface-antigen-directed approaches, including the DLL3 program discussed below.

### 4.3. Clinical Translation and cfDNA Subtyping

Despite these challenges, subtype-guided trials are now in progress. SWOG S2409 (PRISM) is one of the first prospective attempts to operationalize SCLC subtyping for treatment selection, stratifying patients on tumor mRNA profile and SLFN11 status to assign different therapeutic regimens [43]. Beyond tissue-based profiling, cfDNA methylation profiling offers a scalable, non-invasive approach to subtyping that captures temporal evolution. Heeke et al. demonstrated that subtype-specific DNA methylation signatures could be reliably detected in plasma cell-free DNA, enabling longitudinal subtype tracking in SCLC patients during therapy [44]. Such approaches may be especially valuable in SCLC, where biopsy material is limited and treatment-induced plasticity makes one-time tissue sampling unreliable.

From a clinical perspective, longitudinal molecular monitoring may better capture subtype plasticity than a single diagnostic biopsy. At treatment failure, tissue re-biopsy and liquid biopsy are increasingly used to identify resistance mechanisms and characterize tumor evolution. Although multi-region sampling can better assess spatial heterogeneity, serial cfDNA methylation profiling may represent a more feasible and scalable approach for tracking subtype dynamics and informing future biomarker-driven therapeutic strategies [38,42,43,44].

Despite growing biological understanding of transcriptomic subtypes, molecular classification of SCLC remains investigational and has not been incorporated into routine clinical decision-making. Early prospective efforts, such as SWOG S2409 (PRISM), are beginning to evaluate subtype-informed treatment strategies [43]; however, most clinical trials continue to enroll unselected SCLC populations because intratumoral heterogeneity and treatment-induced transcriptional plasticity complicate stable subtype assignment [38,42]. Consequently, subtype-driven therapeutic approaches remain exploratory and hypothesis-generating rather than practice-defining [43].

Molecular subtyping has substantially advanced our understanding of SCLC biology by revealing distinct transcriptional programs, immune states, and therapeutic vulnerabilities that increasingly inform translational research and prospective clinical trial design, supporting continued efforts toward subtype-informed therapeutic strategies [12,13,36,43].

## 5. Predictive Biomarkers in SCLC

The biomarker gap in SCLC is well-documented and remains a major unmet need. Unlike NSCLC, where actionable driver mutations (EGFR, ALK, ROS1, BRAF, KRAS G12C) guide treatment selection, SCLC treatment decisions are made largely without molecular stratification [10]. Below, we review the predictive biomarkers most relevant to current and emerging therapies.

### 5.1. Predictive Biomarkers for Immunotherapy

Emerging data suggest that immune responsiveness in SCLC varies by subtype, with SCLC-A and SCLC-N generally exhibiting less immunogenic phenotypes, while SCLC-I and, in some cohorts, SCLC-P demonstrate more inflamed tumor microenvironments and appear to derive greater benefit from ICI therapy [39,40]. PD-L1 expression on tumor cells is typically low (5–17% of tumors), and responses to ICIs occur regardless of PD-L1 status; PD-L1 has consistently failed to predict immunotherapy benefit in exploratory analyses from IMpower133, CASPIAN, and KEYNOTE-604 [25]. TMB showed initial promise in CheckMate 032, where patients in the highest TMB tertile (≥248 mutations) had better outcomes with nivolumab ± ipilimumab, but subsequent prospective studies failed to validate TMB as a reliable predictive biomarker, and bTMB was not predictive of benefit in IMpower133 [10,22].

### 5.2. SLFN11

Schlafen 11 (SLFN11), a DNA/RNA helicase expressed in approximately 51–76% of SCLC tumors, sensitizes cells to platinum, topoisomerase inhibitors, and PARP inhibitors by triggering replication block and cell death under DNA damage [45,46,47]. Its epigenetic silencing is associated with chemoresistance and worse prognosis. SLFN11 has the strongest mechanistic rationale of any candidate predictive biomarker in SCLC, and its expression status is now being incorporated into prospective trials including SWOG S2409 (PRISM) [43,45]. Clinical translation faces several challenges: the need for standardized immunohistochemistry cutoffs, the dynamic nature of SLFN11 expression under therapy, and the heterogeneity of SLFN11 status within individual tumors. Liquid biopsy-based approaches to longitudinal SLFN11 monitoring are under investigation [47].

Despite these limitations, the high prevalence of SLFN11 expression and its strong mechanistic association with sensitivity to DNA-damaging therapies make it one of the most clinically promising predictive biomarkers currently under evaluation in SCLC [43,45,46,47].

### 5.3. MYC, BCL-2, and Other Vulnerabilities

MYC paralog amplification (c-MYC, MYCN, MYCL) occurs in approximately 20–30% of SCLC tumors and is associated with aggressive biology, NEUROD1 lineage bias, and poor outcomes [4,5,6]. MYC-driven tumors exhibit dependencies on Aurora kinase A and CHK1, and several MYC-targeted strategies (RRx-001, MRT-2359) are under investigation [5,7]. BCL-2 overexpression is enriched in SCLC-A tumors and represents an additional therapeutic vulnerability, although BH3-mimetic clinical development in SCLC has so far been mixed [7]. Dammert et al. showed that MYC paralog–dependent apoptotic priming orchestrates a spectrum of vulnerabilities across SCLC subtypes, providing a rationale for subtype-stratified BCL-2- and CHK1-directed strategies [6].

Additional biomarkers are emerging in SCLC, including epigenetic regulators such as PRC2/EZH2 and alterations in chromatin regulators, as well as IFN-γ-related gene signatures, STING signaling, MHC expression, and tertiary lymphoid structures, all of which may further refine patient selection and improve understanding of antitumor immunity and therapeutic responsiveness [38,39,40,42].

## 6. DLL3 as a Case Study: Same Target, Two Modalities, Opposite Outcomes

### 6.1. DLL3 Biology and Rationale

DLL3 is an atypical member of the Notch ligand family. Unlike the canonical ligands DLL1, DLL4, JAG1, and JAG2, DLL3 lacks the DSL-domain sequences required to productively activate Notch signaling in trans. In normal tissue, it is expressed primarily in the developing nervous system and adult testis, with minimal expression in other adult tissues [37]. In SCLC and other high-grade neuroendocrine tumors, DLL3 is induced downstream of the lineage transcription factor ASCL1 and is expressed on the tumor-cell surface in approximately 70–96% of SCLC cases overall, although expression levels vary considerably. Intermediate-to-high DLL3 expression is observed in roughly two-thirds of tumors and is particularly enriched in ASCL1-driven (SCLC-A) and NEUROD1-driven (SCLC-N) subtypes [29,37,48]. Mechanistically, DLL3 acts in cis to inhibit Notch signaling within the same cell, reinforcing the neuroendocrine phenotype that defines most SCLC. Its aberrant surface localization in tumor cells, combined with its near absence in normal tissues, made DLL3 an attractive candidate for antibody-based targeting from its initial characterization [37]. Importantly, DLL3 expression is concentrated in ASCL1-driven (SCLC-A) and NEUROD1-driven (SCLC-N) tumors, with substantially lower expression in POU2F3 (SCLC-P) and inflamed (SCLC-I) subtypes—a subtype dependence with direct consequences for target coverage and a point we return to in Section 8 [13,26].

### 6.2. Rovalpituzumab Tesirine: A Validated Target, a Failed Drug

Rovalpituzumab tesirine (rova-T) was developed by Stemcentrx (later AbbVie) as a humanized IgG1 anti-DLL3 antibody conjugated via a cleavable peptide linker to a pyrrolobenzodiazepine (PBD) dimer DNA cross-linking payload [37]. Preclinical data were striking: rova-T eliminated tumor-initiating cells in patient-derived xenografts and produced durable regressions in SCLC models [37]. The first-in-human phase 1 study enrolled 74 patients with recurrent SCLC and reported a confirmed response rate of 18% overall and 38% among patients with high DLL3 expression (≥50% of tumor cells), generating substantial enthusiasm for a disease with few second-line options [14] Table 3.

Three larger confirmatory studies followed. The phase 2 TRINITY study evaluated rova-T as third-line or later therapy in patients with DLL3-expressing SCLC [15]. The objective response rate (ORR) by independent review was approximately 12%, with no meaningful enrichment by DLL3 expression level. Median OS was approximately 5.6 months. Serositis (pleural and pericardial effusions), photosensitivity reactions, peripheral edema, and thrombocytopenia were common [15]. TAHOE compared rova-T to topotecan as second-line therapy in DLL3-high SCLC and was halted early following an interim analysis for an unfavorable risk–benefit profile, with inferior survival in the rova-T arm [16]. MERU evaluated rova-T as maintenance therapy after first-line platinum–etoposide and was terminated for futility, with no PFS or OS benefit [17].

The consistent finding across these trials was not that DLL3 was the wrong target. Tumor biopsies confirmed expression. Responses occurred, including durable ones. Rather, the drug’s therapeutic index was inadequate: PBD-dimer-associated toxicity—capillary-leak-like serositis, hepatic injury, and cutaneous reactions—constrained dosing and duration, while the payload’s dependence on efficient internalization, trafficking, and accumulated DNA damage may have been mismatched to a tumor population with rapid turnover and heterogeneous antigen density. Rova-T reached the tumor; what it delivered, and at what cost, did not add up.

### 6.3. Tarlatamab: Same Target, Different Modality, Different Outcome

Tarlatamab (AMG 757) is a half-life-extended BiTE consisting of an anti-DLL3 single-chain variable fragment (scFv) linked to an anti-CD3 scFv, fused to an Fc domain that extends serum half-life [52]. Unlike an ADC, tarlatamab carries no cytotoxic payload. Its mechanism is to bring a cytotoxic T cell into functional contact with a DLL3-expressing tumor cell, driving T-cell activation, granzyme and perforin release, and serial killing [29,52].

The first-in-human DeLLphi-300 study established feasibility, pharmacokinetics, and a recommended phase 2 dose, with responses observed across DLL3 expression levels and in heavily pretreated patients [18]. The pivotal phase 2 DeLLphi-301 study randomized 222 patients with relapsed or refractory SCLC across 10 mg and 100 mg intravenous dose cohorts [19]. At the 10 mg dose ultimately selected for approval, the confirmed ORR was 40%, with a median duration of response of 9.7 months and median PFS of 4.9 months. More recently, a post hoc intracranial analysis of the phase III DeLLphi-304 trial demonstrated meaningful CNS activity with tarlatamab. Among patients with baseline brain metastases, tarlatamab improved median CNS progression-free survival compared with chemotherapy (6.5 vs. 4.2 months; HR 0.40), achieved CNS tumor shrinkage of ≥30% in 56% of evaluable patients versus 38% with chemotherapy, and produced CNS complete responses in 15% versus 5% of patients, respectively. Median overall survival among patients with baseline brain metastases was also prolonged with tarlatamab (13.9 vs. 6.8 months; HR 0.51), supporting clinically meaningful intracranial efficacy in both treated and untreated asymptomatic brain metastases [53]. These values are clinically meaningful for a disease whose second-line cytotoxics typically produce response rates of 15–35% and response durations of 3–5 months [19,21]. Cytokine release syndrome (CRS) occurred in approximately half of treated patients (mostly grade 1–2 with step-up dosing), and immune effector cell-associated neurotoxicity syndrome (ICANS) or ICANS-like neurological events occurred in approximately 8–10% [19]; both are mechanistically expected for a T-cell engager and are generally manageable with structured monitoring in early cycles.

Tarlatamab initially received FDA accelerated approval in May 2024, followed by traditional FDA approval in November 2025 [21] Table 3. The confirmatory phase 3 DeLLphi-304 trial subsequently demonstrated a significant survival benefit over investigator-choice chemotherapy as second-line therapy in 509 patients with progression after platinum-based chemotherapy: median OS was 13.6 versus 8.3 months (HR 0.60; 95% CI 0.47–0.77; *p* < 0.001), supporting tarlatamab as a new standard of care in this setting [20]. The critical point for this review is not that tarlatamab is dramatically superior in response rate to chemotherapy—its ORR is of similar magnitude to that of lurbinectedin or topotecan—but that it produces durable responses in a disease historically characterized by brief, uniform relapses, and it does so against the same target that rova-T failed to exploit. The key variable that changed was the therapeutic modality.

### 6.4. Modality as the Decisive Variable: Mechanistic Interpretation

Why would two drugs against the same antigen produce opposite outcomes? Several features of SCLC biology map onto the mechanistic differences between ADCs and T-cell engagers.

**Figure 2:**
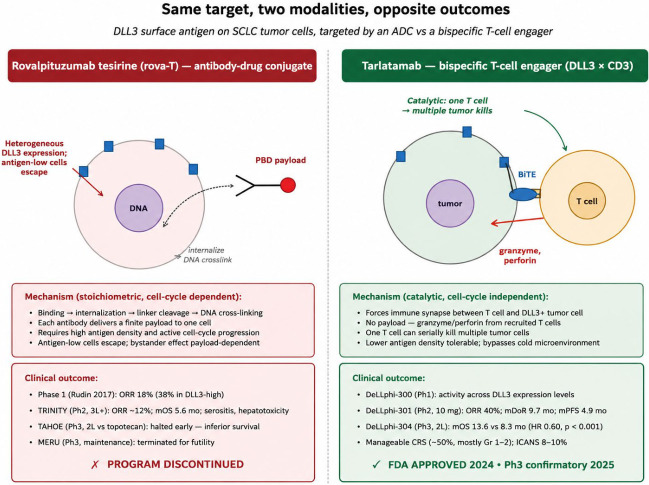
DLL3 case study—same target, two modalities, opposite outcomes. Rovalpituzumab tesirine (rova-T; **left**) is an antibody-drug conjugate that delivers a pyrrolobenzodiazepine (PBD) DNA-cross-linking payload after binding, internalization, and linker cleavage. Each antibody molecule delivers a finite payload to one cell, and cytotoxicity depends on adequate antigen density and active cell-cycle progression. TRINITY (phase II single-arm), TAHOE, and MERU failed, and the program was discontinued. Tarlatamab (**right**) is a half-life-extended BiTE that bridges DLL3 on the tumor cell to CD3 on a cytotoxic T cell, forcing an immune synapse independent of endogenous priming. Cytotoxicity is catalytic (one T cell can serially kill multiple tumor cells), payload-free, and cell-cycle-independent. Tarlatamab received FDA accelerated approval in 2024 (DeLLphi-301) and subsequently achieved traditional FDA approval in 2025 following demonstration of a survival benefit over chemotherapy in the confirmatory phase 3 DeLLphi-304 trial. The variable that changed between failure and success was the delivery modality, not the target.

- **Antigen heterogeneity.** SCLC tumors show intratumoral heterogeneity in DLL3 expression, with cells ranging from high-expressing to antigen-negative within the same tumor [20,22,29]. ADCs are stoichiometric: each bound antibody delivers a finite payload to the cell it binds. Low-expressing and antigen-negative cells escape, and the bystander effect from membrane-permeable payloads is variable and payload-dependent. T-cell engagers are catalytic—a single activated T cell can serially kill multiple tumor cells, and the engaged T cell can recruit additional T cells through cytokine release and epitope spreading. Heterogeneous expression is more forgiving when the effector is immunological.
- **Proliferation kinetics.** SCLC has among the highest proliferation rates of any solid tumor. ADCs with DNA-damaging payloads depend on sufficient drug accumulation, cell-cycle progression, and the inability to repair damage before division. A tumor that doubles rapidly can outpace subtherapeutic intracellular payload concentrations. BiTE-mediated cytotoxicity does not depend on tumor-cell division: granzyme-mediated apoptosis occurs within hours of immune synapse formation [22].
- **Immune context.** SCLC generally exhibits a less immunogenic tumor microenvironment with low T-cell infiltration, factors that have limited the magnitude of checkpoint inhibitor benefit despite high TMB [54]. BiTEs force immune engagement independent of endogenous priming—they manufacture a synapse where none would otherwise have existed. They bypass, rather than depend on, the pre-existing immune landscape.
- **Payload toxicity.** PBD dimers have a steep toxicity curve; their off-target effects (serositis, hepatotoxicity, cutaneous toxicity) constrained rova-T dosing throughout development [15,16,17]. Newer ADC payloads—particularly topoisomerase I inhibitors such as deruxtecan (DXd)—have wider therapeutic windows and more controlled bystander effects, a point directly relevant to the B7-H3 program discussed in Section 7 [27,55]. Notably, the failure of rovalpituzumab tesirine should not be interpreted as a failure of the ADC modality itself. Whereas Rova-T employed a PBD payload with a narrow therapeutic window and substantial toxicity, newer ADCs incorporating DXd payloads have demonstrated broader therapeutic indices and encouraging activity, suggesting that payload engineering may be as important as target selection in determining ADC success in SCLC [14,15,16,17,27,55]. The mechanistic contrast between rova-T and tarlatamab and their corresponding clinical outcomes is summarized in Figure 2.

Additional DLL3-directed approaches, including obrixtamig (BI 764532), trispecific T-cell engagers such as HPN328, CAR-T therapies, and radioligand strategies, are currently in development. Furthermore, combination strategies incorporating DLL3-targeted therapies with chemotherapy (topotecan) or immunotherapy may ultimately prove more effective than monotherapy; this suggests that therapeutic success in SCLC is multifactorial and depends on the interplay between target biology, therapeutic modality, tumor heterogeneity, and treatment context [29,48,50,51].

## 7. Beyond DLL3: B7-H3 and SEZ6 as Emerging Surface-Antigen Therapeutic Programs

If the DLL3 story generalizes, other surface antigens in SCLC should show the same pattern: success contingent on modality choice, with BiTEs succeeding broadly and ADCs succeeding when payload and linker have been engineered to match the disease.

The emphasis on DLL3 in this review should not be interpreted as exclusivity of therapeutic relevance but rather as a biologically informative model through which broader principles of target validation and modality selection can be examined. The increasing clinical development of B7-H3- and SEZ6-directed therapies underscores that multiple surface antigens may ultimately contribute to precision treatment strategies in SCLC [26,27,55].

### 7.1. B7-H3 (CD276)

B7-H3 (CD276), a transmembrane immune checkpoint protein, is expressed in 65–80% of SCLC cases, often with greater homogeneity than DLL3, and—critically—across multiple transcription factor subtypes including POU2F3 [26]. Gay et al. profiled B7-H3 RNA expression in 1721 SCLC tumors and demonstrated consistently high expression across SCLC-A, -N, -P, and -I subtypes, whereas DLL3 and SEZ6 expression each varied significantly by subtype [26]. This subtype-agnostic expression pattern is biologically meaningful: it predicts that a B7-H3-directed agent should retain target coverage even when SCLC tumors undergo subtype transitions under therapy, an advantage DLL3-directed therapies lack Table 4.

Ifinatamab deruxtecan (I-DXd; DS-7300) is an ADC combining an anti-B7-H3 antibody with a cleavable tetrapeptide linker and a topoisomerase I inhibitor payload (DXd)—the same payload class that has transformed HER2-targeted therapy via trastuzumab deruxtecan [55]. In the IDeate-Lung01 phase 2 trial in previously treated ES-SCLC, I-DXd at the 12 mg/kg dose produced an ORR of approximately 55% with a manageable toxicity profile dominated by gastrointestinal and hematologic events and a small but real rate of interstitial lung disease [26,27]. The agent has progressed to phase 3 evaluation in IDeate-Lung02 against investigator-choice chemotherapy, and the BLA received FDA Priority Review in 2026 [27]. Additional B7-H3-directed agents, including risvutatug rezetecan (HS-20093/GSK5764227), have also demonstrated encouraging activity in relapsed ES-SCLC, supporting B7-H3 as an increasingly relevant therapeutic target while underscoring the need for continued characterization of hematologic toxicity and interstitial lung disease across this therapeutic class [56].

Notably, ifinatamab deruxtecan has also demonstrated encouraging intracranial activity in patients with baseline brain metastases. In the primary analysis of IDeate-Lung01, the intracranial confirmed objective response rate (cORR) was 46.2%, with an intracranial disease control rate of 90.8%; among patients with baseline target brain lesions, the intracranial cORR reached 65.5%. These findings suggest meaningful CNS activity and support further prospective evaluation of this agent in patients with SCLC and brain metastases [57]. If these findings hold, they will suggest that the ADC modality in SCLC is viable once the payload problem that doomed rova-T is solved—a refinement of, rather than a refutation of, the modality thesis. The wider therapeutic index of DXd-class payloads, combined with the more homogeneous B7-H3 expression pattern, addresses both the toxicity and antigen-coverage problems simultaneously.

### 7.2. SEZ6

Seizure-related homolog protein 6 (SEZ6) is a type 1 transmembrane protein with adult tissue expression largely restricted to the central nervous system, aberrantly overexpressed in SCLC and other high-grade neuroendocrine carcinomas [49]. SEZ6 expression is regulated by ASCL1 and is therefore enriched in SCLC-A and SCLC-N, with expression patterns broadly comparable to DLL3 [57]. ABBV-011 (anti-SEZ6 ADC bearing a calicheamicin payload) has shown early clinical activity in relapsed SCLC, with delayed hepatotoxicity prompting dose reduction during early development. ABBV-706, a topoisomerase I-payload anti-SEZ6 ADC, is in early-phase clinical evaluation [49] Table 4. Like B7-H3, SEZ6 represents a test of whether a more forgiving payload class can rescue the ADC modality in a disease that punished PBD-payload ADCs.

For several other emerging surface-antigen–directed therapies, including DLL3-directed trispecific T-cell engagers (HPN328), obrixtamig (BI 764532), SEZ6-targeted ADCs, CAR-T approaches, and DLL3 radioligand programs, the intracranial activity of these agents remains largely undefined because prospective CNS-specific analyses have not yet been performed.

## 8. Limits of the Modality-First Frame

Any clean thesis in oncology is a target for its own counterexamples, and the modality-first frame has real limits that merit explicit statement.

First, tarlatamab’s response rate is 40%, not 80%. Sixty percent of DLL3-expressing tumors do not respond. Target expression is necessary but evidently not sufficient, and the biology of primary and acquired resistance to DLL3 BiTEs—likely involving antigen loss, HLA downregulation, T-cell exhaustion, and subtype plasticity—remains poorly characterized. The modality changed the drug’s outcome but did not make the target universally exploitable.

Second, subtype plasticity is a real threat to all surface-antigen-directed therapies. SCLC tumors can shift from ASCL1-high to NEUROD1-high phenotypes under therapeutic pressure, and POU2F3 or inflamed subtypes express DLL3 poorly [13,42]. A patient whose tumor undergoes a subtype switch may lose the antigen regardless of modality. Combinations that either prevent plasticity or target antigens expressed across subtypes (B7-H3 being the leading candidate) may be required.

Third, toxicity is not free. CRS and ICANS with tarlatamab require inpatient monitoring for initial step-up dosing, with real implications for access and patient selection in community practice. Although BiTEs such as tarlatamab have demonstrated meaningful efficacy in relapsed SCLC, their administration requires careful logistical planning due to the risk of CRS and ICANS. In our institution, implementation has involved standardized order sets, patient education, trained infusion staff, pharmacy coordination, access to corticosteroids and tocilizumab, and clear escalation pathways involving neurology, inpatient observation, and intensive care support when needed. Therefore, community adoption may require referral pathways or hub-and-spoke models, particularly for early step-up dosing and monitoring. The modality that succeeds biologically may still face logistical constraints. Bispecific T-cell engagers are also subject to the same general resistance mechanisms that limit cellular immunotherapy in solid tumors—antigen-negative escape, T-cell exhaustion in the tumor microenvironment, and on-target/off-tumor toxicities tied to even low-level expression in normal tissues.

Fourth, and most importantly, the thesis does not claim that any surface antigen plus the right modality will produce a drug. The claim is narrower: when a target has been validated and a drug fails, modality is an underappreciated explanation and deserves explicit consideration before the target itself is abandoned. This reframing has direct practical implications for ongoing development decisions: programs that fail with one modality should not necessarily be abandoned but reconsidered with alternative delivery mechanisms whose biology better matches the disease’s constraints.

## 9. Conclusions and Future Directions

**Figure 3:**
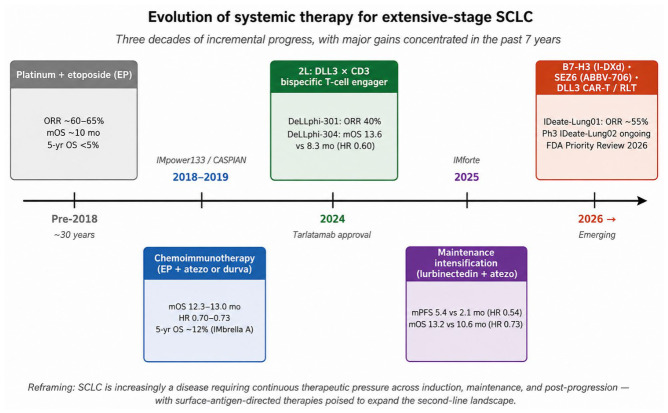
Evolution of systemic therapy for extensive-stage SCLC. Platinum-etoposide remained the standard for approximately three decades, with a median OS near 10 months. The addition of PD-L1 inhibitors to platinum-etoposide (IMpower133, CASPIAN) produced the first major OS gain in a generation, with hazard ratios of 0.70–0.73 and a small but reproducible long-term-survivor tail. Tarlatamab (DeLLphi-301/-304) established a new second-line standard of care, culminating in traditional FDA approval in 2025 following positive confirmatory phase 3 results. IMforte (2025) introduced maintenance intensification with lurbinectedin plus atezolizumab. Emerging surface-antigen-directed programs targeting B7-H3 (ifinatamab deruxtecan), SEZ6 (ABBV-706), and next-generation DLL3 modalities (CAR-T, radioligand therapy) are positioned to further expand the post-progression landscape over the coming years.

The treatment of ES-SCLC has undergone its most substantial transformation in a generation (Figure 3). Chemoimmunotherapy with atezolizumab- or durvalumab-based regimens, established by IMpower133 and CASPIAN, produced consistent improvements in overall survival with hazard ratios of 0.70–0.73 [8,9], a meaningful expansion of long-term survivors at five years [24], and—most recently—an option for maintenance intensification with lurbinectedin plus atezolizumab in IMforte [11]. These advances are real but limited: most patients still relapse within months, and the absence of validated predictive biomarkers leaves treatment selection essentially empirical.

The molecular framework of SCLC—four transcriptomic subtypes with distinct lineage transcription factor programs, immune microenvironments, and surface-antigen expression patterns—provides the substrate for subtype-guided precision oncology. Real-world data have refined the framework: most SCLC tumors are mixed, or transcription factor-negative, intratumoral heterogeneity is the rule rather than the exception, and subtypes shift under therapy. cfDNA methylation profiling and prospective subtype-stratified trials such as SWOG S2409 (PRISM) are operationalizing what for years remained a retrospective taxonomic exercise.

The DLL3 story sharpens what target discovery in this disease should mean. The same validated surface antigen, pursued first with a failed ADC and then with a successful BiTE—now supported by positive phase 3 data and traditional FDA approval in second-line ES-SCLC [20,21]—reframes the central question. The bottleneck in SCLC drug development has not been the absence of druggable surface biology. It has been the mismatch between target biology and delivery modality. Rapid proliferation, antigen heterogeneity, subtype plasticity, and a less immunogenic microenvironment penalize modalities that depend on cell-autonomous dependency or efficient payload accumulation, and they reward modalities that recruit catalytic, cell-cycle-independent cytotoxic effectors. The emerging B7-H3 (ifinatamab deruxtecan) and SEZ6 (ABBV-706) programs, together with next-generation DLL3 modalities including CAR-T and radiopharmaceuticals, will test whether the framework generalizes [27,55,56,57].

An additional implication of the modality framework is that future therapeutic success in SCLC will likely depend on rational combination strategies rather than prolonged single-agent approaches. The current therapeutic paradigm already incorporates chemotherapy plus immune checkpoint inhibition, and maintenance intensification with lurbinectedin plus atezolizumab has further improved outcomes in selected patients. Similarly, ongoing studies are evaluating DLL3-directed therapies in combination with chemotherapy and immunotherapy in earlier treatment settings. Given the biological complexity of SCLC, combinations that simultaneously exploit surface-antigen targeting, engage antitumor immunity, and address subtype-specific vulnerabilities may ultimately provide deeper and more durable clinical benefit than any individual modality alone.

The practical implication for the field is that improving outcomes in SCLC will likely require an integrated approach combining molecular subtype characterization, predictive biomarker development, rational target selection, and therapeutic modality optimization. For a disease long described as undruggable, therapeutic success will probably depend not on any single target or platform, but on adaptive, multimodal, and biomarker-informed strategies capable of addressing tumor evolution and therapeutic resistance.

## Figures and Tables

**Table 1:** Pivotal phase 3 first-line trials in extensive-stage SCLC.

Trial	Design/N	Regimen	Efficacy	Key Caveats/Biomarker Findings
IMpower133 [8,10,22]	Randomized, double-blind Ph1/3; N = 403	Atezolizumab + carboplatin/etoposide → atezo maintenance vs. placebo + EP	mOS 12.3 vs. 10.3 mo (HR 0.70; *p* = 0.007); mPFS 5.2 vs. 4.3 mo (HR 0.77); 5-yr OS ~12% (IMbrella A)	PD-L1 and bTMB not predictive; durable benefit confined to a small long-term tail
CASPIAN [9,23]	Open-label Ph3; N = 805	Durvalumab ± tremelimumab + EP (cis or carbo) vs. EP	mOS 13.0 vs. 10.3 mo (HR 0.73); 3-yr OS 17.6% vs. 5.8%	Tremelimumab arm not statistically superior; included asymptomatic brain mets; no biomarker predicted benefit
IMforte [11]	Randomized open-label Ph3 maintenance; post-induction	Lurbinectedin + atezolizumab vs. atezolizumab maintenance (after EP + atezo induction)	mPFS 5.4 vs. 2.1 mo (HR 0.54); mOS 13.2 vs. 10.6 mo from start of maintenance (HR 0.73; *p* = 0.0174)	OS measured from maintenance start (not induction); progressors during induction excluded—population enriched for favorable biology

Abbreviations: atezolizumab (atezo); blood tumor mutational burden (bTMB); etoposide-platinum (EP); hazard ratio (HR); median overall survival (mOS); median progression-free survival (mPFS); overall survival (OS); progression-free survival (PFS).

**Table 2:** Molecular subtypes of SCLC and their therapeutic implications.

Subtype	Defining TF	Prevalence (Puri et al.) [36]	Surface Antigen Pattern	Immune Phenotype	Therapeutic Vulnerabilities	Refs.
SCLC-A	ASCL1	~26%	DLL3 frequently high (ASCL1-driven); SEZ6 high; B7-H3 high	Less immunogenic with relatively limited T-cell infiltration	DLL3-directed BiTEs/CAR-T; BCL-2 (BH3-mimetics); SEZ6 ADCs	[12,13,26,27,36,37]
SCLC-N	NEUROD1	~10%	DLL3 frequently high; SEZ6 expressed; B7-H3 high	Less immunogenic and characterized by MYC-driven aggressive biology	DLL3-directed therapies; Aurora kinase A; CHK1; MYC pathway	[5,6,7,12,13,26,36]
SCLC-P	POU2F3	~4%	DLL3 low/negative; SEZ6 low; B7-H3 high	Variable; tuft-cell-like, non-neuroendocrine	PARP inhibitors; nucleotide metabolism; B7-H3 ADCs	[12,13,26,36]
SCLC-I	Inflamed (low TF)	subset within TF-low (~19.5%) and mixed (~27.9%) tumors	Variable DLL3; B7-H3 broadly retained	IFN-γ signature; antigen presentation high; greatest ICI benefit	Immune checkpoint blockade; combinations exploiting inflamed TME	[13,26,38,39,40]

Abbreviations: antibody-drug conjugate (ADC); B-cell lymphoma 2 (BCL-2); bispecific T-cell engager (BiTE); chimeric antigen receptor T-cell (CAR-T); checkpoint kinase 1 (CHK1); delta-like ligand 3 (DLL3); immune checkpoint inhibitor (ICI); interferon-gamma (IFN-γ); seizure-related homolog protein 6 (SEZ6); transcription factor (TF); tumor microenvironment (TME).

**Table 3:** Selected DLL3-directed agents and outcomes by modality.

Agent	Modality	Pivotal Trial(s)	Outcome	Status
Rovalpituzumab tesirine (rova-T)	ADC (PBD payload)	TRINITY, TAHOE, MERU	ORR ~12%; OS not improved; halted for toxicity/futility	Discontinued [14,15,16,17]
Tarlatamab (AMG 757)	BiTE (DLL3 × CD3)	DeLLphi-300, -301, -304	ORR 40%; mDoR 9.7 mo; mOS 13.6 vs. 8.3 mo (Ph3, HR 0.60)	FDA approved 2025; Ph3 confirmatory 2025 [18,19,20,21]
BI 764532 (obrixtamig)	BiTE	Phase 1 (dose escalation)	Early-phase activity reported	Ongoing [29,48]
HPN328	Trispecific (DLL3/CD3/albumin)	Phase 1/2	Early-phase activity reported	Ongoing [29,48]
ABBV-011	ADC (calicheamicin payload)	Phase 1	Limited data; hepatotoxicity dose-limiting	Ongoing/limited [49]
DLL3-targeted CAR-T (incl. VHH-based, IL-18-secreting)	CAR-T	Preclinical/phase 1	Robust preclinical activity; clinical data pending	Early-phase [50]
[177Lu]Lu-DLL3 radioligand therapy	Radioimmunoconjugate	Preclinical (translational)	Imaging and therapy feasibility demonstrated in preclinical and pilot human studies	Translational [51]

Abbreviations: antibody-drug conjugate (ADC); bispecific T-cell engager (BiTE); chimeric antigen receptor T-cell (CAR-T); cytokine release syndrome (CRS); immune effector cell-associated neurotoxicity syndrome (ICANS); median duration of response (mDoR); median overall survival (mOS); objective response rate (ORR); overall survival (OS); phase (Ph); pyrrolobenzodiazepine (PBD).

**Table 4:** Emerging surface-antigen-directed therapeutic strategies beyond DLL3 in SCLC.

Target	Representative Agent	Modality	Development Stage	Key Findings	Refs.
B7-H3	Ifinatamab deruxtecan (I-DXd)	ADC (DXd payload)	Phase III	ORR approximately 55%; manageable toxicity profile; preliminary CNS activity	[26,27,55]
SEZ6	ABBV-011	ADC (calicheamicin payload)	Phase I	Early antitumor activity; delayed hepatotoxicity requiring dose optimization	[49]
SEZ6	ABBV-706	ADC (topoisomerase I payload)	Phase I	Ongoing evaluation, designed to improve therapeutic index	[49]
DLL3	HPN328	Trispecific T-cell engager	Phase I/II	Early clinical activity reported	[29,48]
DLL3	VHH-based and IL-18-secreting CAR-T products	CAR-T	Early phase	Robust preclinical efficacy; clinical development ongoing	[50]
DLL3	[177Lu] Lu-DLL3	Radioligand therapy	Translational	Demonstrated feasibility for imaging and targeted radiotherapy	[51]

Abbreviations: antibody-drug conjugate (ADC); chimeric antigen receptor T-cell (CAR-T); delta-like ligand 3 (DLL3); deruxtecan payload (DXd); ifinatamab deruxtecan (I-DXd); objective response rate (ORR); seizure-related homolog protein 6 (SEZ6).

## Data Availability

No new data were created or analyzed in this study. Data sharing is not applicable.

## Abbreviations

ADC, antibody–drug conjugate; ASCL1, achaete-scute homolog 1; bTMB, blood tumor mutational burden; BiTE, bispecific T-cell engager; CAR-T, chimeric antigen receptor T-cell; cfDNA, cell-free DNA; CRS, cytokine release syndrome; DLL3, delta-like ligand 3; DXd, deruxtecan payload; EP, etoposide–platinum; ES-SCLC, extensive-stage small cell lung cancer; HR, hazard ratio; ICANS, immune effector cell-associated neurotoxicity syndrome; ICI, immune checkpoint inhibitor; I-DXd, ifinatamab deruxtecan; mOS, median overall survival; mPFS, median progression-free survival; NE, neuroendocrine; NEUROD1, neurogenic differentiation 1; NSCLC, non-small cell lung cancer; ORR, objective response rate; PBD, pyrrolobenzodiazepine; PD-1, programmed death 1; PD-L1, programmed death-ligand 1; POU2F3, POU class 2 homeobox 3; rova-T, rovalpituzumab tesirine; SCLC, small cell lung cancer; SEZ6, seizure-related homolog protein 6; SLFN11, Schlafen 11; TMB, tumor mutational burden; YAP1, yes-associated protein 1.
